# Metabolic and Fatigue Profiles Are Comparable Between Prepubertal Children and Well-Trained Adult Endurance Athletes

**DOI:** 10.3389/fphys.2018.00387

**Published:** 2018-04-24

**Authors:** Anthony Birat, Pierre Bourdier, Enzo Piponnier, Anthony J. Blazevich, Hugo Maciejewski, Pascale Duché, Sébastien Ratel

**Affiliations:** ^1^AME2P, UFR STAPS, Clermont-Auvergne University, Clermont-Ferrand, France; ^2^Centre for Exercise and Sports Science Research, School of Exercise and Health Sciences, Edith Cowan University, Joondalup, WA, Australia; ^3^French Rowing Federation, Nogent-sur-Marne, France

**Keywords:** growth, aerobic metabolism, high-intensity exercise, young people, recovery

## Abstract

The aim of this study was to determine whether prepubertal children are metabolically comparable to well-trained adult endurance athletes and if this translates into similar fatigue rates during high-intensity exercise in both populations. On two different occasions, 12 prepubertal boys (10.5 ± 1.1 y), 12 untrained men (21.2 ± 1.5 y), and 13 endurance male athletes (21.5 ± 2.7 y) completed an incremental test to determine the power output at VO_2max_ (PVO_2max_) and a Wingate test to evaluate the maximal anaerobic power (P_max_) and relative decrement in power output (i.e., the fatigue index, FI). Furthermore, oxygen uptake (VO_2_), heart rate (HR), and capillary blood lactate concentration ([La]) were measured to determine (i) the net aerobic contribution at 5-s intervals during the Wingate test, and (ii) the post-exercise recovery kinetics of VO_2_, HR, and [La]. The P_max_-to-PVO_2max_ ratio was not significantly different between children (1.9 ± 0.5) and endurance athletes (2.1 ± 0.2) but lower than untrained men (3.2 ± 0.3, *p* < 0.001 for both). The relative energy contribution derived from oxidative metabolism was also similar in children and endurance athletes but greater than untrained men over the second half of the Wingate test (*p* < 0.001 for both). Furthermore, the post-exercise recovery kinetics of VO_2_, HR, and [La] in children and endurance athletes were faster than those of untrained men. Finally, FI was comparable between children and endurance athletes (−35.2 ± 9.6 vs. −41.8 ± 9.4%, respectively) but lower than untrained men (−51.8 ± 4.1%, *p* < 0.01). To conclude, prepubertal children were observed to be metabolically comparable to well-trained adult endurance athletes, and were thus less fatigable during high-intensity exercise than untrained adults.

## Introduction

It has been widely demonstrated that prepubertal children fatigue less than untrained adults when performing dynamic, whole-body activities such as maximal cycling (Ratel et al., 2002), short running bouts (Ratel et al., 2004), and vertical jumps (Lazaridis et al., 2018), or maximum voluntary contractions under isometric (Hatzikotoulas et al., 2014; Ratel et al., 2015) or isokinetic conditions (De Ste Croix et al., 2009). The lower fatigability in prepubertal children has been mainly attributed to a lower peripheral (i.e., muscular) fatigue compared to untrained adults, owing to their greater relative reliance on oxidative energy sources (Ratel et al., 2008; Tonson et al., 2010) and their potentially greater proportion of fatigue-resistant slow-twitch muscle fibers (Lexell et al., 1992). This specific metabolic profile in prepubertal children could lead to a lower accumulation of metabolic by-products (e.g., H^+^ ions and inorganic phosphate) and a lower phosphocreatine depletion during high-intensity exercise in prepubertal children compared to untrained adults (Kappenstein et al., 2013). As these metabolic by-products promote the development of peripheral fatigue through an alteration of contractile properties and reduction in excitation-contraction coupling (Allen et al., 2008), a lower accumulation of metabolites could translate into a reduced fatigue at the periphery, as usually observed in prepubertal children (Hatzikotoulas et al., 2014; Ratel et al., 2015).

Beyond muscular factors, prepubertal children also display faster O_2_ uptake and heart rate (HR) recovery kinetics following high-intensity exercise than untrained adults (Armon et al., 1991; Hebestreit et al., 1993). This could be ascribed to their lower anaerobic capacity and thus to their smaller O_2_ deficit incurred at the start of the exercise, which calls for a lesser excess post-exercise O_2_ consumption. Furthermore, prepubertal children could be characterized by a greater parasympathetic reactivation of the autonomic nervous system early in the recovery period after exercise than untrained adults (Ohuchi et al., 2000). Taken together, this could explain why prepubertal children are able to complete repeated high-intensity exercise bouts easier when compared to their older untrained counterparts (Ratel et al., 2002, 2004).

However, it is now known that metabolic profiles show significant inter-individual variability in young adults, and is specifically associated with their training background. While adults with a strong sprint training background predominantly rely on anaerobic energy support during exercise, endurance athletes depend more notably on oxidative energy pathways than either untrained adults or sprint-trained athletes (Pesta et al., 2013). This more oxidative profile in endurance athletes translates particularly into (i) a faster recovery of power output (Bogdanis et al., 1995) and cardio-respiratory parameters following high-intensity exercise (Short and Sedlock, 1997) and (ii) a lower peripheral fatigue during repeated maximum voluntary contractions (Garrandes et al., 2007). Hence, it is possible that metabolic and fatigue profiles of well-trained adult endurance athletes could be similar to those observed in prepubertal children during high-intensity exercise, as previously stated by Ratel and Blazevich (2017). However, agreement has not been reached because there is a lack of scientific evidence relating to this issue.

Therefore, the aim of the present study was to determine whether, contrary to untrained adults, prepubertal children are metabolically comparable to well-trained adult endurance athletes and if this translates into similar fatigue rates during high-intensity exercise in both populations. We hypothesized that the relative energy contribution derived from oxidative metabolism during high-intensity exercise would be similar in prepubertal children and endurance-trained adult athletes, and this could lead to comparable fatigue rates between both populations. Furthermore, it is presumed that untrained adults rely less on oxidative metabolism and therefore fatigue faster during high-intensity exercise than prepubertal children and well-trained adult endurance athletes.

## Materials and methods

### Participants

Twelve healthy boys (age: 8–12 y), 12 untrained men (19–23 y), and 13 endurance male athletes (19–27 y) volunteered to participate in the study. To be included, boys and untrained men had to perform recreational physical activity for ≤4 h per week and to be free of any medical contra-indication to physical activity. Boys were prepubertal based on the assessment of their somatic maturity (see below). None of them were involved in any vigorous physical activity or engaged in a specific aerobic training program. Boys were recruited from primary and secondary schools while untrained men were university students. Their recreational physical activities were Alpine skiing, snowboarding, sailing, skateboarding, climbing, etc. In contrast, endurance-trained adults were engaged in long distance physical activities for ≥6 times a week for at least 2 years and were national-level competitive athletes (i.e., long-distance runners, cyclists, and triathletes). They were recruited from local sports clubs (athletics, triathlon, and cycling). This study was approved by a local Institutional Ethics Review Board. The study was conducted in conformity with the policy statement regarding the use of human subjects by the Declaration of Helsinki. All experimental procedures were clearly explained to the participants, who then gave written consent before the commencement of testing. Written consent was also obtained from parents/guardians before children were accepted into the study.

### Experimental procedure (design)

All participants were tested in two experimental sessions separated by at least 48 h. During the first experimental session, anthropometric characteristics, body composition, maturation status, and the power at maximum O_2_ uptake (PVO_2max_) were evaluated. Furthermore, the participants had to perform two sprints (~7 s) on a cycle ergometer (Cyclus model II, MSE Electronic Medical, Leipzig, Germany) separated by 1 min of recovery against a resistance corresponding to 7.5% body mass (BM). These sprints served to familiarize the volunteers with the experimental procedures of the second session. During the second visit, the participants were asked to perform a Wingate cycle test to determine the maximal anaerobic power (P_max_), the fatigue rate (i.e., the relative decrement in power output), the relative (net) energy contribution derived from oxidative metabolism and the post-exercise recovery rates of blood lactate concentration, HR, and O_2_ uptake. The Wingate cycle test was chosen because it allows the simultaneous investigation of cardio-respiratory, metabolic, and muscular components of fatigue during a whole-body dynamic activity and is a common and well-learned activity within the population. Furthermore, the Wingate cycle test was found to be highly reliable from one session to another in children and adults for the evaluation of high-intensity exercise performance indices (Hebestreit et al., 1993).

### Anthropometric measurements and body composition analysis

Body mass was measured to the nearest 0.1 kg using a digital weight scale (TANITA, BC-545N, Japan) and standing height was assessed using a portable stadiometer with the participants barefoot (TANITA, HR001, Japan). Sitting height was also measured with the stadiometer while the participants sat on the floor with their back against a wall. Body Mass Index (BMI) was subsequently calculated by the equation BM (kg)/height^2^ (m^2^). Skinfold thickness was measured in duplicate at the triceps and subscapular sites using a Harpenden caliper (Baty International, Burgess Hill, UK). The measurements were taken by the same investigator on the right side of the body to reduce variability in the results. Body fat (BF, %) was assessed using Slaughter's equations (Slaughter et al., 1988). Body fat (kg) was calculated by multiplying BF (%) by BM (kg). Fat-free mass (FFM, kg) was determined by subtracting BF (kg) from BM (kg).

### Maturation assessment

Age from peak height velocity (APHV) was used to assess somatic maturity and determined by using height, sitting height and BM. Its calculation was only done in children and was based on sex-specific regression equations according to the method proposed by Mirwald et al. (2002).

### Maximal aerobic power assessment

PVO_2max_ was assessed using a submaximal graded test on a cycle ergometer (Cyclus model II, MSE Electronic Medical, Leipzig, Germany). Workloads were initially set at 30, 100, and 130 W and then increased by 15, 30, and 30 W every 3 min in prepubertal children, untrained adults, and well-trained adult endurance athletes, respectively. The pedaling rate was constant and self-selected by the participants between 50 and 90 rpm. The test was designed to have one warm-up stage and three to four additional submaximal stages leading HR to at least 160 bpm. HR was measured during the last minute of each stage using the Polar recorder linked to the cycle ergometer (Polar Electro, Kempele, Finland). If the participant's HR did not reach a plateau or the participant did not maintain cadence, the measurement was considered to be invalid. PVO_2max_ was then assessed from individual linear regressions between power output and HR and by calculating the power output at the age-predicted maximal HR (HR_max_). The squared Bravais-Pearson correlation coefficients of these linear relationships ranged between 0.91 and 0.99 (mean ± SD: 0.98 ± 0.02). HR_max_ was assessed using the equation formulated by Shargal et al. (2015), which was validated from a large cohort of healthy males aged between 10 and 80 years:

(1) $$\begin{array}{r} \text{H}{\text{R}}_{\text{max}}=208.609-0.716\times \text{age} \end{array}$$

Furthermore, VO_2max_ was assessed using the following equation:

(2) $$\begin{array}{r} \text{V}{\text{O}}_{2\text{max}}\left({\text{L}\cdot \text{min}}^{-1}\right)=\left(\text{PV}{\text{O}}_{2\text{max}}\left(\text{W}\right)\times 60\right)/\left(\text{E}\times \text{R}\right) \end{array}$$

where *E* is the caloric equivalent of oxygen for a respiratory exchange ratio above 1.0, i.e., 21131 J·L^−1^, and *R* the cycling efficiency (%). A delta efficiency of 23% was chosen for all the volunteers since the literature reports no apparent difference during cycling between 10.5-year-old boys (23.2%), 21.3-year-old untrained men (22.5%), and 25.6-year-old well-trained endurance triathletes and cyclists (22.7%) (Rowland et al., 1990; Louis et al., 2012).

### The fatigue protocol

The testing session started with a rest period of 10 min in a sitting position on the cycle ergometer. The saddle height was set at 107% of trochanteric leg length to give optimal comfort to each participant (Hamley and Thomas, 1967). Afterwards, the participants were asked to perform a standardized warm-up of 4 min cycling against a resistance that was adjusted to 2% of their BM. At the end of the second, third, and fourth minutes, they also had to perform one sprint lasting 5 s against a resistance corresponding to 7.5% BM. After a subsequent 5-min rest, the participants were instructed to perform a Wingate test that consisted of pedaling as fast as possible for 30 s against a resistance corresponding to 0.075 g·kg^−1^ BM (Bar-Or, 1987). This braking load was chosen as it does not appear to influence P_max_ or fatigue rate during the Wingate test, when compared to heavier loads (e.g., 11% BM), in either untrained adults or less-powerful athletes such as endurance athletes (Jaafar et al., 2016). Furthermore, it corresponds to an optimal braking load to produce P_max_ and mean power output (P_mean_) during a Wingate test in children aged from 6 to 12 years (Carlson and Naughton, 1994). Prior to each sprint, the experimenter announced a countdown of “5-4-3-2-1-GO” during which the participants were instructed to put the right crank arm 45° past top position. After test termination, the subjects were supervised during a 20-min recovery period in a sitting position. The participants were instructed not to engage in any strenuous activity on the day preceding the exercise test.

#### Ratings of perceived exertion

Ratings of perceived exertion (RPE) were noted immediately after completion of the Wingate test using the Children's Effort Rating Table (Williams et al., 1994). The Children's Effort Rating Table (CERT) asks participants to rate their effort on a 1–10 scale with 1 indicating that the exercise is very, very easy, and 10 indicating that the effort is so hard that the participant has to stop. Before testing, the range of sensations that correspond to categories of effort within the scale were clearly explained to each participant.

#### Blood sampling and analysis

Capillary blood samples (0.2 μL) were obtained from the fingertips prior to the warm-up ([La]_rest_), before the Wingate test, immediately after completion of the test ([La]_(0)_), and at 1, 3, 5, 7, 9, 15, and 20 min in recovery. Blood lactate concentrations were determined using the Lactate Scout^+^ analyzer (EKF Diagnostic, Leipzig, Germany). The device was systematically calibrated before each experimental session.

#### Gas exchange and HR recording

During the second experimental session, HR was continuously monitored using the Polar recorder linked to the Cyclus Model II ergometer (Polar Electro, Kempele, Finland). VO_2_ was also continuously measured breath-by-breath using a mobile spiroergometry system (METAMAX®3B, CORTEX Biophysik GmbH, Leipzig, Germany).

### Data processing

#### Power and fatigue indices

Maximal anaerobic power (P_max_) and minimal power (P_min_) reached during the Wingate test were defined as the highest and the lowest mechanical powers respectively, recorded over a 0.5-s period. The average power was considered as the mean power output (P_mean_) sustained over the whole 30-s test. The fatigue index (FI) was calculated as the difference between P_max_ and P_min_ expressed as percentage of P_max_ (Bar-Or, 1987). The maximum anaerobic-to-aerobic mechanical power ratio was calculated by normalizing the Wingate test-derived P_max_ to the estimated maximal aerobic power (P_max_-to-PVO_2max_ ratio). This anaerobic-to-aerobic power ratio was introduced by Bar-Or (1986) as a simple metabolic index to assess physiological function. A higher power ratio indicates a higher anaerobic ability relative to aerobic capacity. Several studies in adult elite athletes (cyclists and swimmers) have also demonstrated the usefulness of this mechanical power ratio in the physiological evaluation of these athletes, as the power ratio gives recommendations as to whether endurance training or specific strength and power training should be emphasized (Mercier et al., 1993; Baron, 2001).

#### Relative energy contribution

The aerobic energy (in J) utilized during the Wingate test was calculated at 5-s intervals based on the net oxygen uptake (VO_2_−VO_2rest_) and the caloric equivalent of oxygen for a respiratory exchange ratio above 1.0, i.e., 21131 J·mL^−1^. The total energy consumed during the Wingate test was assessed from the mechanical work done at 5-s intervals and by considering a cycling efficiency of 23% for all the volunteers (Rowland et al., 1990; Louis et al., 2012). The relative energy contribution (%) derived from aerobic metabolism was then calculated at 5-s intervals throughout the Wingate test.

#### HR and VO_2_ recovery kinetics

Resting VO_2_ and HR values (VO_2rest_ and HR_rest_, respectively) were determined in a sitting position during the first 10 min of the second experimental session. Furthermore, the peak values of VO_2_ and HR (VO_2pk_ and HR_pk_, respectively) were considered as the highest values reached at the end of the Wingate test. As resting and peak values of VO_2_ and/or HR differed between groups, the post-exercise recovery kinetics of VO_2_ and HR were determined by considering the net changes (peak exercise–baseline) expressed as percentage of peak values. Subsequent comparisons were done from values taken at 15-s intervals during the first 2-min and at 60-s intervals during the last 8-min. Values were more frequently taken at first in consideration of the initial fast phase of recovery of VO_2_ and HR in subsequent comparisons between groups.

#### [La] recovery curves

Individual blood lactate recovery curves were fitted to the biexponential time function:

(3) [La](t)=[La](0)+A1(1−e−γt1)+A2(1−e−γt2)

where [La]_(0)_ and [La](*t*) (mmol·L^−1^) are lactate concentrations in capillary blood measured at recovery onset and at a given recovery time, respectively; concentration parameters A_1_ and A_2_ (mmol.L^−1^) are the amplitudes of the exponential functions, and γ_1_ and γ_2_ (min^−1^) are the velocity constants that describe lactate exchange and removal capacity, respectively (Zouloumian and Freund, 1981). The blood lactate recovery curves were fitted to eq. 3 by iterative nonlinear regression using the GraphPad Prism 7 software (La Jolla, California, USA) to determine the values of A_1_, A_2_, γ_1_, and γ_2_; [La]_(0)_ being an experimental measurement. Furthermore, the highest blood lactate concentration reached during the post-exercise recovery period ([La]_pk_) and the time required to reach [La]_pk_ (*t*[La]_pk_) were calculated from individual blood lactate recovery curves. Equation (3) was considered representative of lactate recovery kinetics since it accounted for more than 96% of the variance in the experimental blood lactate recovery curves (0.965 < *r* < 0.998).

### Statistical analysis

Data were screened for normality of distribution and homogeneity of variances using a Shapiro-Wilk normality test and the Barlett's test, respectively. One-way (group) ANOVA was used to compare age, anthropometric characteristics, performance outcomes (e.g., P_max_, PVO_2max_, P_max_-to-PVO_2max_ ratio) and blood lactate kinetics parameters (e.g., A_1_, A_2_, γ_1_, γ_2_, La]_pk_, *t*La]_pk_) between groups. When ANOVA revealed a significant effect, a Newman-Keuls *post*-*hoc* test was applied to test the discrimination between means. Furthermore, a two-way (group × time) ANOVA with repeated measures was used to analyze the relative energy contribution from oxidative metabolism throughout the Wingate test as well as the post-exercise recovery kinetics of HR and O_2_ uptake. When ANOVA revealed a significant main or interaction effect, a Newman-Keuls *post*-*hoc* test was applied to test the discrimination between means. The size effect and statistical power have also been reported when significant main or interaction effects were detected. The size effect was assessed using the partial eta-squared (η^2^) and ranked as follows: ~0.01 = small effect, ~0.06 = moderate effect, ≥0.14 = large effect (Cohen, 1969). A linear regression model between the fatigue index and the P_max_-to-PVO_2max_ ratio was fitted by the least squares method considering all the volunteers, and a squared Bravais-Pearson correlation coefficient (*r*^2^) of this linear regression model was calculated. The limit for statistical significance was set at *p* < 0.05. Statistical procedures were performed using Statistica 8.0 software (Statsoft, Inc., USA). Results were presented in the text, tables and figures as mean ± SD.

## Results

### Participants' physical characteristics

The physical characteristics of participants are presented in Table 1. ANOVA revealed significant differences between the different groups for age [*F*_(2, 34)_ = 131.1, *p* < 0.001, η^2^ = 0.88, power = 1.0], height [*F*_(2, 34)_ = 110.2, *p* < 0.001, η^2^ = 0.87, power = 1.0], BM [*F*_(2, 34)_ = 123.2, *p* < 0.001, η^2^ = 0.88, power = 1.0], BMI [*F*_(2, 34)_ = 61.2, *p* < 0.001, η^2^ = 0.78, power = 1.0], BF (%) [*F*_(2, 34)_ = 20.4, *p* < 0.001, η^2^ = 0.55, power = 0.99], FFM (kg) [*F*_(2, 34)_ = 183.8, *p* < 0.001, η^2^ = 0.92, power = 1.0], HR_rest_ [*F*_(2, 34)_ = 33.6, *p* < 0.001, η^2^ = 0.66, power = 1.0], PVO_2max_ (W) [*F*_(2, 34)_ = 118.4, *p* < 0.001, η^2^ = 0.87, power = 1.0], PVO_2max_/BM (W·kg^−1^) [*F*_(2, 34_) = 26.1, *p* < 0.001, η^2^ = 0.61, power = 0.99], and the P_max_-to-PVO_2max_ ratio [*F*_(2, 34)_ = 38.5, *p* < 0.001, η^2^ = 0.69, power = 1.0]. *Post*-*hoc* tests showed no significant difference for age and height between untrained adults and well-trained adult endurance athletes. However, untrained adults displayed significantly higher values for BM (*p* < 0.05), BMI (*p* < 0.01), BF (*p* < 0.05), FFM (*p* < 0.05), HR_rest_ (*p* < 0.001) when compared to their well-trained endurance counterparts. In contrast, as expected, PVO_2max_ (W and W·kg^−1^) was significantly higher in well-trained adult endurance athletes than untrained adults (*p* < 0.001 for both).

**Table 1:** Participants' physical characteristics.

	**C**	**EA**	**UA**
	***n* = 12**	***n* = 13**	***n* = 12**
Age (y)	10.5 ± 1.1	21.5 ± 2.7^***^	21.2 ± 1.5^***^
Years to (from) APHV	−3.2 ± 1.0	–	–
Height (cm)	141.6 ± 7.5	177.4 ± 7.5^***^	178.3 ± 5.6^***^
BM (kg·m^−2^)	32.9 ± 5.1	67.5 ± 7.0^***^	75.3 ± 8.7^***^^$^
BMI (kg·m^−2^)	16.3 ± 1.1	21.4 ± 1.5^***^	23.7 ± 2.3^***^^$$^
BF (%)	16.4 ± 2.9	8.7 ± 2.0^***^	11.3 ± 4.0^***^^$^
FFM (kg)	27.5 ± 4.2	61.6 ± 6.0^***^	66.6 ± 5.9^***^^$^
HR_rest_ (bpm)	81.8 ± 8.6	53.2 ± 7.5^***^	72.7 ± 10.6^***^^$$$^
VO_2max_/BM (mL·min^−1^·kg^−1^)	49.0 ± 7.9	67.1 ± 6.9^***^	48.1 ± 7.7^$$$^
PVO_2max_ (W)	130.2 ± 26.9	366.2 ± 46.3^***^	290.5 ± 40.2^***^^$$$^
PVO_2max_/BM (W·kg^−1^)	4.0 ± 0.6	5.4 ± 0.6^***^	3.9 ± 0.6^$$$^
P_max_-to-PVO_2max_ ratio	1.9 ± 0.5	2.1 ± 0.2	3.2 ± 0.3^***^^$$$^

*Values are means ± SD. C, children; EA, endurance athletes; UA, untrained adults*.

*, **, *** *Significantly different from children at p < 0.05, p < 0.01 and p < 0.001, respectively*.

$, $$, $$$ *Significantly different from endurance athletes (EA) at p < 0.05, p < 0.01 and p < 0.001, respectively. APHV, age at the peak height velocity; BM, body mass; BMI, body mass index; BF, body fat; FFM, fat-free mass; HR, heart rate; VO_2max_, maximum oxygen uptake; PVO_2max_, power at maximum oxygen uptake*.

Furthermore, stature, BM, BMI, FFM, and PVO_2max_ (W) were significantly lower in prepubertal children than untrained adults and well-trained adult endurance athletes (*p* < 0.001 for each). In contrast, prepubertal children displayed significantly higher values for BF and HR_rest_ than their older trained and untrained counterparts (*p* < 0.05 at least). However, PVO_2max_/BM (W·kg^−1^) was not significantly different between prepubertal children and untrained adults.

Interestingly, no significant difference was observed in the P_max_-to-PVO_2max_ ratio between prepubertal children and well-trained adult endurance athletes; however, the P_max_-to-PVO_2max_ ratio was significantly higher in untrained adults than prepubertal children and well-trained adult endurance athletes (*p* < 0.001 for both) (Table 1).

### Exercise period

**Figure 1:**
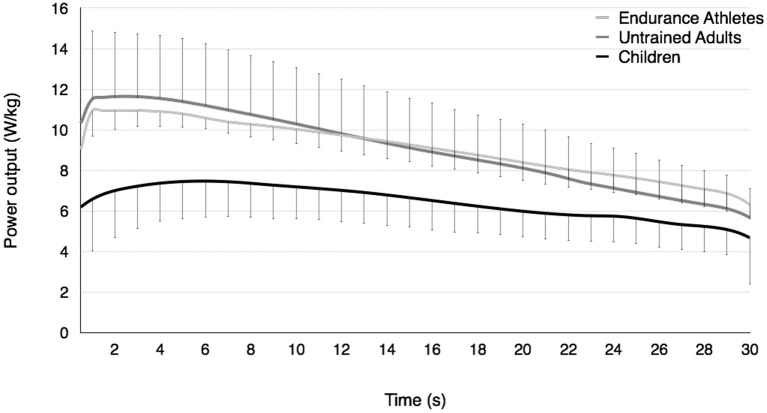
Time course of power output during the Wingate test in prepubertal children, untrained adults, and well-trained adult endurance athletes. The relative decrement in power output (i.e., the fatigue index) is higher in untrained adults than children and well-trained adult endurance athletes but similar in children and well-trained adult endurance athletes.

Performance outcomes obtained during the Wingate test are displayed in Figure 1 and Table 2. ANOVA revealed significant differences between the groups for P_max_ (W) [*F*_(2, 34)_ = 178.8, *p* < 0.001, η^2^ = 0.91, power = 1.0], P_max_/BM (W·kg^−1^) [*F*_(2, 34)_ = 49.6, *p* < 0.001, η^2^ = 0.74, power = 1.0], *t*P_max_ [*F*_(2, 34)_ = 12.4, *p* < 0.001, η^2^ = 0.42, power = 0.99], P_mean_ (W) [*F*_(2, 34)_ = 201.4, *p* < 0.001, η^2^ = 0.92, power = 1.0], P_mean_/BM (W·kg^−1^) [*F*_(2, 34)_ = 46.9, *p* < 0.001, η^2^ = 0.73, power = 1.0], FI [*F*_(2, 34)_ = 12.6, *p* < 0.01, η^2^ = 0.43, power = 0.99], and RPE [*F*_(2, 34)_ = 3.7, *p* < 0.05, η^2^ = 0.18, power = 0.64]. In contrast, ANOVA showed no significant effect for HR_pk_ between the groups.

**Table 2:** Performance outcomes during the Wingate test in prepubertal children, untrained adults and well-trained adult endurance athletes.

	**C**	**EA**	**UA**
	***n* = 12**	***n* = 13**	***n* = 12**
**POWER OUTPUT**			
P_max_ (W)	247.5 ± 74.1	771.3 ± 105.1^***^	905.2 ± 87.5^***^^$$$^
P_max_/BM (W·kg^−1^)	7.5 ± 1.4	11.4 ± 1.0^***^	12.1 ± 1.3^***^
tP_max_ (s)	5.8 ± 3.3	1.9 ± 1.6^***^	1.8 ± 1.6^***^
P_mean_ (W)	207.1 ± 44.5	612.1 ± 73.0^***^	678.9 ± 65.6^***^^$^
P_mean_/BM (W·kg^−1^)	6.3 ± 0.8	9.1 ± 0.7^***^	9.1 ± 0.9^***^
FI (%)	35.2 ± 9.6	41.8 ± 9.4	51.8 ± 4.1^***^^$$^
**RATINGS OF PERCEIVED EXERTION**			
RPE (1–10 scale)	7.5 ± 1.0	8.5 ± 1.1^*^	8.1 ± 0.7^*^
**HEART RATE**			
HR_pk_ (bpm)	175.5 ± 12.4	166.6 ± 13.0	175.0 ± 6.3

*Values are means ± SD. C,children; EA, endurance athletes; UA, untrained adults*.

*, **, *** *Significantly different from children at p < 0.05, p < 0.01, and p < 0.001, respectively*.

$, $$, $$$ *Significantly different from endurance athletes at p < 0.05, p < 0.01, and p < 0.001, BM, body mass; P_max_, maximal anaerobic power; tP_max_, time required to reach; P_max_, P_mean_, mean power output; FI, Fatigue index; RPE, rating of perceived exertion; HR_pk_, peak heart rate*.

More specifically, untrained adults displayed significantly higher values for P_max_ (W), P_mean_ (W), and FI (%) than well-trained adult endurance athletes (*p* < 0.05 at least). However, P_max_/BM (W·kg^−1^), P_mean_/BM (W·kg^−1^), tP_max_, and RPE were not significantly different between untrained and endurance trained adults. Furthermore, children displayed significantly lower values than untrained and trained adults for all parameters investigated (*p* < 0.05 at least) except for FI (%), which was similar to that of endurance-trained adults.

**Figure 2:**
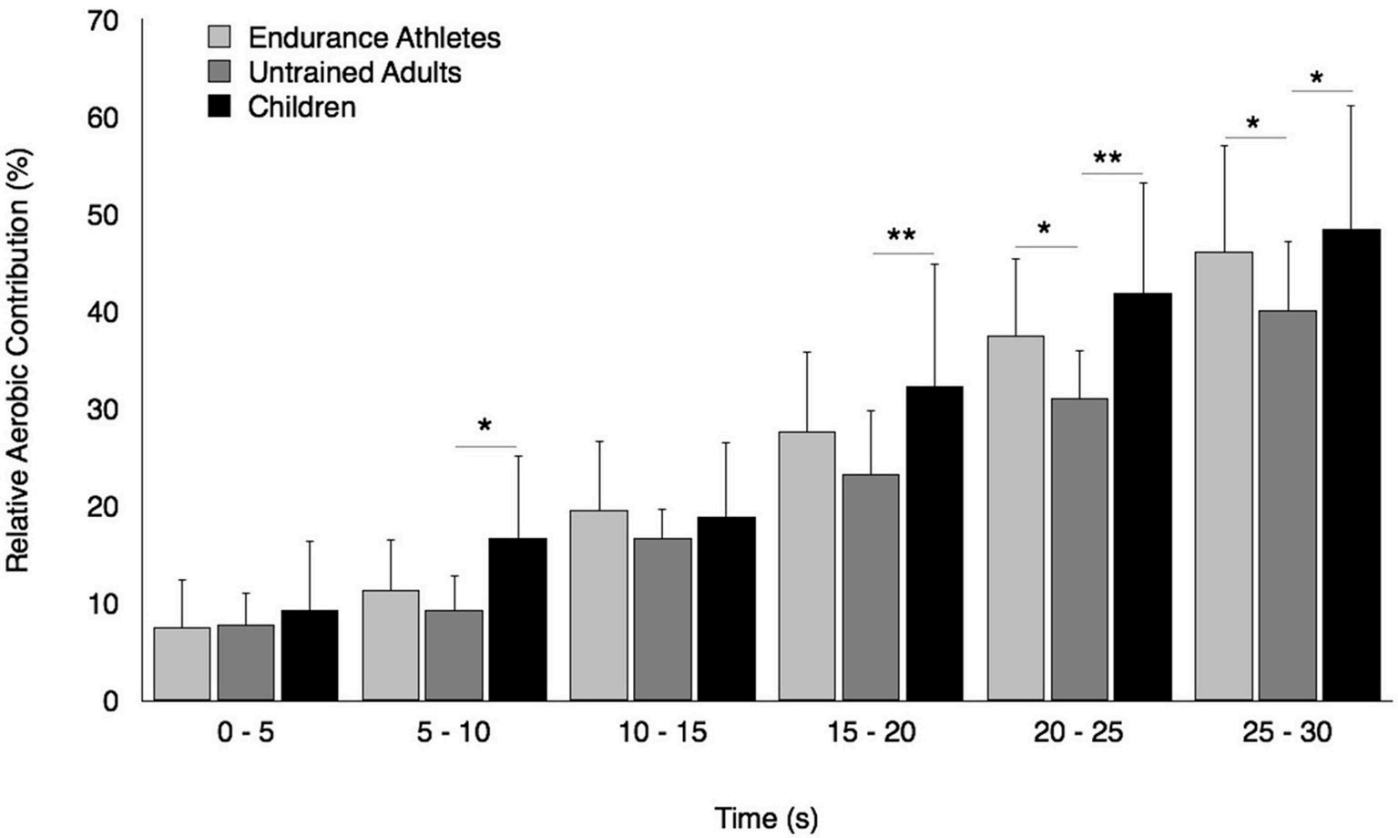
Relative contribution of energy derived oxidative metabolism at 5-s intervals over the Wingate test in prepubertal children, untrained adults, and well-trained adult endurance athletes. ^*^, ^**^Significantly different at *p* < 0.05 and *p* < 0.01, respectively.

Regarding the relative aerobic contribution, ANOVA tended to show a group × time interaction effect [*F*_(10, 170)_ = 3.86, *p* = 0.09, η^2^ = 0.09, power = 0.78]. The relative aerobic contribution increased progressively during the Wingate test whatever the group considered (Figure 2). However, this increment was not significantly different between prepubertal children and well-trained adult endurance athletes but significantly lower in untrained adults compared to prepubertal children over the second half of the Wingate test (*p* < 0.05 at least). Furthermore, well-trained adult endurance athletes displayed a significantly greater relative aerobic contribution than untrained adults over the last 10 s of the Wingate test (*p* < 0.05).

### Post-exercise recovery period

**Figure 3:**
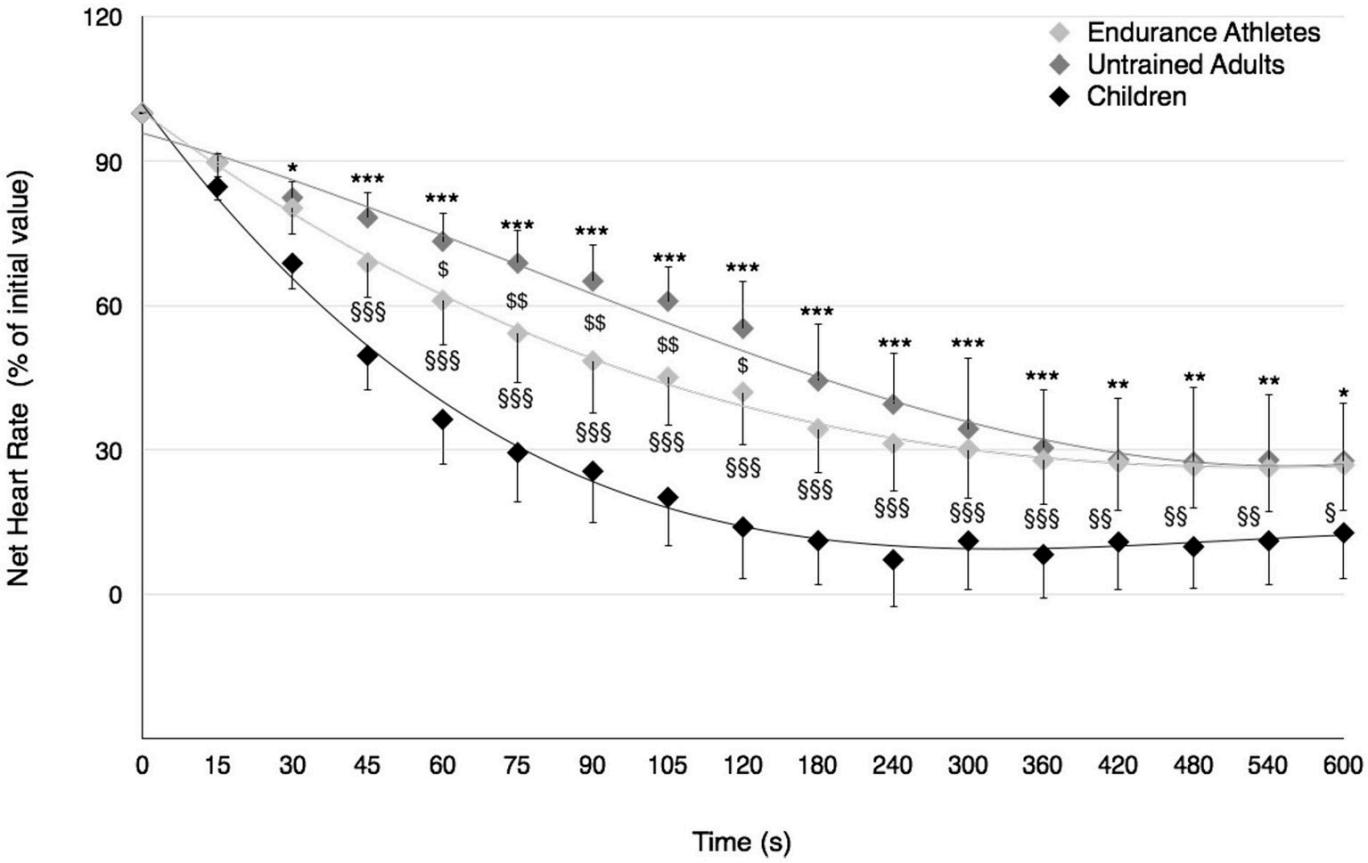
Recovery of net heart rate after the Wingate test in prepubertal children, untrained adults, and well-trained adult endurance athletes. Values were expressed as percentage of initial values. ^*^, ^**^, ^***^Significantly different at *p* < 0.05, *p* < 0.01, and *p* < 0.001, respectively, between children and untrained adults. ^*$, $$*^Significantly different at *p* < 0.05 and *p* < 0.01, respectively, between untrained adults and well-trained adult endurance athletes. ^§, §§, §§§^Significantly different at *p* < 0.05, *p* < 0.01, and *p* < 0.001, respectively, between children and well-trained adult endurance athletes.

ANOVA revealed a significant group × time interaction throughout the post-exercise recovery period for HR [*F*_(32, 544)_ = 11.71, *p* < 0.001, η^2^ = 0.41, power = 1.0] and VO_2_ [*F*_(32, 480)_ = 2.24, *p* < 0.001, η^2^ = 0.13, power = 0.99]. *Post*-*hoc* tests showed a faster recovery of HR in prepubertal children compared to trained adults from 45-s of recovery, and in prepubertal children compared to untrained adults from 30-s of recovery until the end of test. Furthermore, the recovery rate of HR was significantly faster in endurance-trained athletes compared to untrained adults between the first and second minutes of recovery (Figure 3).

**Figure 4:**
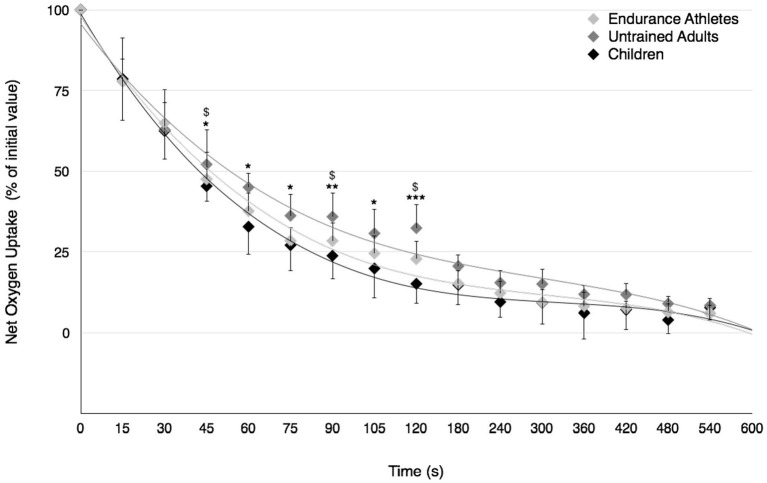
Recovery of net O_2_ uptake after the Wingate test in prepubertal children, untrained adults and well-trained adult endurance athletes. Values were expressed as percentage of initial values. ^*^, ^**^, ^***^Significantly different at *p* < 0.05, *p* < 0.01, and *p* < 0.001, respectively, between children and untrained adults. ^*$*^Significantly different at *p* < 0.05 between untrained adults and well-trained adult endurance athletes.

No significant difference in the recovery rate of VO_2_ was observed between prepubertal children and well-trained adult endurance athletes (Figure 4). However, VO_2_ recovered faster in prepubertal children and well-trained adult endurance athletes compared to untrained male adults between 45 and 120-s of recovery.

Finally, ANOVA revealed a significant group effect for [La]_(0)_ [*F*_(2, 34)_ = 5.85, *p* < 0.01, η^2^ = 0.26, power = 0.84], [La]_pk_ [*F*_(2, 34)_ = 39.88, *p* < 0.001, η^2^ = 0.70, power = 1.0], *t*[La]_pk_ [*F*_(2, 34)_ = 4.81, *p* < 0.05, η^2^ = 0.22, power = 0.76], A_1_ [*F*_(2, 34)_ = 10.08, *p* < 0.001, η^2^ = 0.37, power = 0.98], A_2_ [*F*_(2, 34)_ = 17.8, p < 0.001, η^2^ = 0.51, power = 0.99], and γ_2_ [*F*_(2, 34)_ = 14.31, *p* < 0.001, η^2^ = 0.46, power = 0.99]. In contrast, no significant group effect was observed for [La]_rest_ and γ_1_ (Table 3). More specifically, A_1_, A_2_, and *t*[La]_pk_ were similar in untrained and trained adults but higher than prepubertal children (*p* < 0.05 at least). Furthermore, γ_2_ and [La]_pk_ were significantly different between groups (γ_2_: children > trained adults > untrained adults; [La]_pk_: untrained adults > trained adults > children).

**Table 3:** Blood lactate kinetics parameters obtained during the recovery period of the Wingate test in prepubertal children, untrained adults and well-trained adult endurance athletes.

	**C**	**EA**	**UA**
	***n* = 12**	***n* = 13**	***n* = 12**
[La]_rest_ (mmol·L^−1^)	1.8 ± 0.6	1.8 ± 0.7	2.0 ± 0.7
[La]_(0)_ (mmol·L^−1^)	4.6 ± 1.0	5.3 ± 1.8	7.0 ± 2.4^***^^,^^$^
A_1_ (mmol·L^−1^)	3.7 ± 1.9	8.9 ± 4.7^***^	8.9 ± 2.3^**^
γ_1_ (min^−1^)	0.78 ± 0.33	0.73 ± 0.40	0.86 ± 0.39
A_2_ (mmol·L^−1^)	−7.5 ± 2.2	−13.6 ± 4.9^***^	−15.3 ± 2.2^***^
γ_2_ (min^−1^)	0.07 ± 0.04	0.04 ± 0.01^**^	0.02 ± 0.01^***^^,^^$^
[La]_pk_ (mmol·L^−1^)	6.6 ± 2.1	10.7 ± 2.2^***^	13.9 ± 1.7^***^^,^^$$$^
*t*[La]_pk_ (min)	2.5 ± 1.1	3.8 ± 1.2^*^	4.2 ± 1.8^*^

*Values are means ± SD. C, children; EA, endurance athletes; UA, untrained adults*.

*, **, *** *: Significantly different from children at p < 0.05, p < 0.01, and p < 0.001, respectively*.

$, $$$ *Significantly different from endurance athletes at p < 0.05 and p < 0.001, [La]_rest_, resting blood lactate concentration; [La]_(0)_, blood lactate concentration at end of exercise; A_1_ and A_2_ are the amplitudes of the exponential functions, respectively γ_1_ and γ_2_ are the velocity constants that describe lactate exchange and removal capacity, [La]_pk_, peak blood lactate concentration; t[La]_pk_, time required to reach [La]_pk_*.

### Correlation

**Figure 5:**
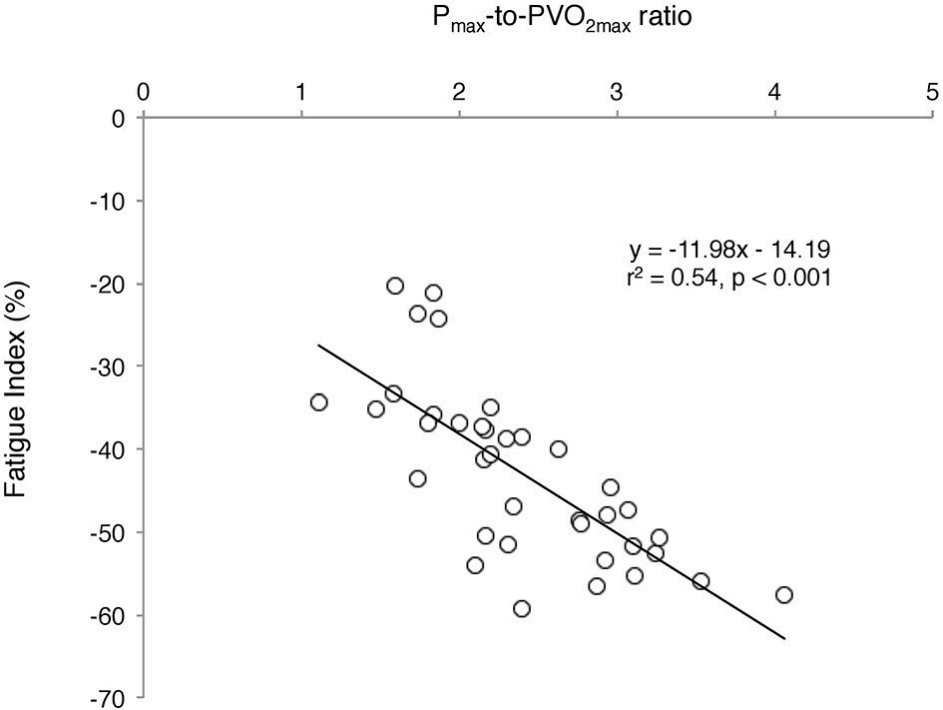
Relationship between the fatigue index (FI, i.e., the relative decrement in power output over the Wingate test) and the maximum anaerobic-to-aerobic mechanical power ratio (P_max_-to-PVO_2max_) considering all participants. The higher the relative anaerobic contribution, the higher the development of fatigue during the Wingate test.

A significant positive relationship was observed between FI (%) and the P_max_-to-PVO_2max_ ratio considering all participants (*r*^2^ = 0.54, *p* < 0.001) (Figure 5).

## Discussion

The aim of the present study was to determine whether, contrary to untrained adults, prepubertal children are metabolically comparable to well-trained adult endurance athletes and if this translates into similar fatigue rates during high-intensity exercise between both populations. The main results confirm our hypotheses since prepubertal children had a comparable net contribution of energy derived from aerobic metabolism to well-trained adult endurance athletes, and the rate of fatigue, as illustrated by the relative decrement in power output during the Wingate test, was similar between both populations. Furthermore, the post-exercise recovery rates of oxygen uptake and HR were respectively similar and faster in prepubertal children than well-trained adult endurance athletes. The removal ability of lactate from the blood compartment was also higher in children than well-trained adult endurance athletes.

### Comparison between children and untrained adults

In the present study, prepubertal children displayed a lower relative decrement in power output during the Wingate test than untrained adults (−35.2 vs. 51.8%, respectively); thus they fatigued much less than their untrained older counterparts. This finding cannot be explained by the fact that children performed more (especially aerobic) physical activity since the boys and men had to meet the same strict inclusion criteria and their relative VO_2max_ were not significantly different (49.0 vs. 48.1 mL·min^−1^·kg^−1^, respectively). The finding is in accordance with the literature showing a reduced susceptibility to fatigue in prepubertal children during different whole-body, high-intensity activities such as cycling (Ratel et al., 2002), running (Ratel et al., 2004), and hopping (Lazaridis et al., 2018), or during maximal voluntary contractions under isometric (Ratel et al., 2015) and isokinetic (De Ste Croix et al., 2009) conditions. The mechanisms underpinning the differences in fatigue between prepubertal children and untrained adults are not yet fully understood but it appears that prepubertal children experience less peripheral (i.e., muscular) fatigue and potentially more central (i.e., neural) fatigue than untrained adults during high-intensity exercise (Streckis et al., 2007; Ratel et al., 2015). The greater fatigue effect on central mechanisms in prepubertal children could account for their lower fatigue at the peripheral level. Indeed, according to one theory, the central nervous system could limit the recruitment of motor units to prevent any extensive homeostasis disturbance, muscle damage, or biological harm (Noakes et al., 2005). However, direct evidence to support this assumption is still lacking. An alternative could be the lesser ability of prepubertal children to fully voluntarily activate motor units during high-intensity exercise (Dotan et al., 2012). Indeed, a lower activation level was found to be associated with a higher resistance to fatigue in adults (Nordlund et al., 2004). In the present study, the maximal voluntary activation level was not measured; however, the longer lag time to reach maximal anaerobic power in children compared to untrained adults (*t*P_max_: 5.8 vs. 1.8 s, respectively; e.g., Figure 1, solid black line) might be suggestive that the amplitude of muscle activation was lower during the early phase of the Wingate test in children and then increased over time, similar to wind-up effects observed in other (e.g., clinical) populations (Hornby et al., 2009). Therefore, the possibility exists that the higher resistance to fatigue in children could be also ascribed to an inability to fully activate the neuromuscular system during the early phase of an explosive exercise bout. This hypothesis should be explicitly examined in future studies.

Among peripheral factors, the greater relative contribution of energy derived from aerobic metabolism in prepubertal children may account for their lower fatigability during high-intensity exercise. The significant positive relationship found in the present study between the fatigue index and the P_max_-to-PVO_2max_ ratio as well as the lower P_max_-to-PVO_2max_ ratio in children compared to young untrained adults (~1.9 vs. 3.2, respectively) support this assertion; the lower the relative anaerobic contribution, the lower the development of fatigue during high intensity exercise in children. Furthermore, the relative oxidative contribution was found to be greater in prepubertal children than untrained adults over the second half of the Wingate test. The recovery kinetics of cardio-respiratory parameters (VO_2_ and HR) and the removal ability of lactate out of the blood compartment (γ_2_) were also faster following the Wingate test in prepubertal children than untrained adults. These results are consistent with previous studies describing the changes in the anaerobic-to-aerobic mechanical power ratio (P_max_-to-PVO_2max_ ratio) during growth using either longitudinal (Falk and Bar-Or, 1993) or cross-sectional (Blimkie et al., 1986; Falgairette et al., 1991) experimental designs, despite aerobic mechanical power not being determined using direct VO_2max_ measurement in the current study. For instance, Falgairette et al. (1991) reported a progressive increment in the P_max_-to-PVO_2max_ ratio until the onset of adolescence (e.g., 1.8 at 9–10 y, 2.2 at 11–12 y, and 2.8 at 14–15 y) in 144 boys, after which it remained stable until early adulthood (~3.0 in physically active but non-competitive young men in the study by Falk and Bar-Or, 1993). Furthermore, some authors have reported (i) a greater relative contribution of energy derived from oxidative metabolism during the Wingate test in 11.8-year-old boys than 16.3-year-old male adolescents (Beneke et al., 2007), and (ii) a smaller O_2_ deficit incurred at the start of cycle exercise at 125% VO_2max_, which called for a lesser excess post-exercise VO_2_ in prepubertal children than untrained adults (Armon et al., 1991). Similarly, the recovery rate of HR was found to be faster in prepubertal children than untrained adults following high-intensity exercise lasting 1 min (Baraldi et al., 1991; Hebestreit et al., 1993). Our results are also consistent with the experimental data derived from blood measurements showing no difference in the exchange capacity of lactate from muscles into blood (γ_1_) between children and untrained adults but a better ability to remove lactate from the blood compartment (γ_2_) in children following the Wingate test (Beneke et al., 2005).

This more oxidative metabolic profile in prepubertal children is usually associated with a lesser accumulation of metabolic by-products (i.e., H^+^ ions, lactate, inorganic phosphate) derived from anaerobic sources in exercising muscle (Kappenstein et al., 2013). In the present study, lactate concentrations were only measured at the blood level, and the bi-exponential time function revealed a lower accumulation with a peak value appearing earlier during the post-exercise recovery period in prepubertal children, as evidenced by their lower A_1_, A_2_, and [La]_pk_ values and shorter *t*[La]_pk_ (Table 3). The underlying mechanisms of this diminished lactate response in prepubertal children has yet to be elucidated, but the pediatric literature often suggests that the lower blood/muscle lactate concentration in prepubertal children results from their lower glycolytic energy turnover (Ratel et al., 2002). Furthermore, it has been shown that the shorter *t*[La]_pk_ in children could be related to their lower accumulated lactate rate during high intensity exercise (Dotan et al., 2003).

While lactate ion accumulation itself has only a small effect on the loss of muscle power in fatigue (Allen et al., 2008), the associated accumulation of metabolic by-products such as H^+^ ions and inorganic phosphate could promote peripheral fatigue to a greater extent through an alteration of contractile processes and the excitation-contraction coupling (Allen et al., 2008). The lesser accumulation of these metabolites in children could therefore translate into a reduced fatigue at the periphery, as usually observed in this population during high-intensity exercise (Hatzikotoulas et al., 2014; Ratel et al., 2015). In addition, the lower anaerobic capacity and the greater parasympathetic reactivation of the autonomic nervous system early in the recovery period after exercise in prepubertal children could explain the faster readjustment of cardiovascular parameters (Armon et al., 1991; Ohuchi et al., 2000) and the higher removal ability of lactate from the blood compartment (γ_2_).

### Comparison between children and endurance adult athletes

In contrast to untrained adults, prepubertal children displayed the same metabolic profile as well-trained adult endurance athletes. The P_max_-to-PVO_2max_ ratio was similar in children and endurance-trained athletes (1.9 vs. 2.1, respectively), and was consistent with those previously reported in well-trained adult endurance athletes who had VO_2max_ values between 60 and 70 mL·min^−1^·kg^−1^ (~2.0 for Meeuwisse et al., 1992; Hostrup et al., 2016). Furthermore, the relative energy contribution derived from oxidative metabolism during the Wingate test as well as the post-exercise recovery rate of VO_2_ were similar in children and endurance adult athletes. On this basis, prepubertal children could be considered analogous to well-trained adult endurance athletes from a physiological perspective, despite them having a lower work capacity than their older counterparts, i.e., a lower mean power output during the Wingate test (Ratel and Blazevich, 2017). Surprisingly, the results of the present study also showed a faster post-exercise HR recovery rate in prepubertal children than endurance adult athletes. This unexpected result could be ascribed to a greater parasympathetic reactivation of the autonomic nervous system in the early recovery period in prepubertal children than well-trained adult endurance athletes, owing to a greater central cholinergic modulation of HR (Ohuchi et al., 2000). However, further studies comparing the low-frequency and high-frequency components of HR variability in the early recovery period between both populations are required to test this assumption. Furthermore, the results of the present study indicated a lack of difference in γ_1_ and a higher γ_2_ in children than well-trained adult endurance athletes, suggesting that prepubertal children could have a better capacity to remove lactate from the blood compartment than their older trained counterparts. This difference in γ_2_ may be related to the shorter circulation time in children (Cumming, 1978), as evidenced by their faster HR recovery rate in the present study; the faster HR recovery rate, the higher lactate removal ability (γ_2_) following the Wingate test.

On a more practical level, the similar metabolic profile of prepubertal children and well-trained adult endurance athletes translated into comparable fatigue rates during the Wingate test (−35.2 vs. −41.8%, respectively) despite children taking more time to reach their maximal anaerobic power output. This is consistent with experimental data obtained by Hebestreit et al. (1993) and Harbili (2015) who indirectly showed comparable fatigue rates during the Wingate test between prepubertal boys (−44%) and professional male road cyclists of Olympic and national levels (−43%). Based on these data, it is presumed that the greater reliance on oxidative energy sources and the lower relative anaerobic contribution in well-trained adult endurance athletes (Pesta et al., 2013) translate into a lower peripheral (i.e., muscular) fatigue, as usually observed in prepubertal children compared to untrained adults during repeated maximal voluntary muscle contractions (Hatzikotoulas et al., 2014; Ratel et al., 2015). However, until now the central and peripheral components of neuromuscular fatigue during high-intensity exercise in well-trained adult endurance athletes have only been compared to those of explosive power-trained athletes (Garrandes et al., 2007) and not to untrained adults. Further studies comparing the peripheral and central components of neuromuscular fatigue between prepubertal children, well-trained adult endurance athletes and untrained adults are therefore required to check this assumption.

## Conclusion

The results of the present study showed a comparable net contribution of energy derived from aerobic metabolism during the Wingate test between prepubertal children and well-trained adult endurance athletes. Furthermore, the post-exercise recovery kinetics of oxygen uptake and HR were respectively similar and faster in prepubertal children than well-trained adult endurance athletes. The removal ability of lactate out of the blood compartment was also higher in children than well-trained adult endurance athletes. These results could explain why the rate and magnitude of fatigue in prepubertal children are similar to well-trained adult endurance athletes and why they recover faster from high-intensity exercise than untrained adults.

## Practical applications

On a more practical level, the results of the present study suggest that prepubertal children may not have to perform specific training to develop their aerobic metabolic competence. Other strategies might be considered before puberty to improve exercise performance, including entrainment of anaerobic systems and movement technique training to improve mechanical efficiency. In contrast, as the maturational and growth processes have an adverse effect on oxidative energy production in exercising muscle, aerobic training may be a high priority in pubertal and post-pubertal children to maintain their aerobic potential and delay the development of exercise-induced fatigue.

## Ethics statement

This study was carried out in accordance with the recommendations of the local Institutional Ethics Review Board. The protocol was approved by the local Institutional Ethics Review Board. All subjects gave written informed consent in accordance with the Declaration of Helsinki.

## Author contributions

All authors contributed to the data analysis and interpretation of the data, drafting, and revising the manuscript, and approved the final version of the manuscript. The original study design was made by AB, PB, AJB, PD, and SR and discussed with the other authors. AB, PB, EP, and HM performed the data analysis.

### Conflict of interest statement

The authors declare that the research was conducted in the absence of any commercial or financial relationships that could be construed as a potential conflict of interest.
